# U. S. V. M. A.

**Published:** 1896-11

**Authors:** 


### U. S. V. M. A.

So much interest has been awakened in the work of the profession, through the meetings of the national association, and so many distinct interests are centred in this body, that the time seems to have arrived when we should discuss the wisdom of section work for a part of the time of the annual convention. As a possible means of arriving at some definite plan for our convention programme for 1897, it has been suggested that the afternoon of the second day shall be devoted to section work, and that the convention divide up into sections such subjects as State Control of Tuberculosis, Experiment-station Work, Practical Papers for Every-day Practitioners, Field of Canine Pathology, etc. The JOURNAL proposes to open its columns for the discussion of this all-important aspect of our association work, believing that such a course will best enable those officers charged with the preparation of the programme to make it more attractive to all, thereby insuring a hearing to every member who shall prepare a paper.

Those members desiring cloth-bound copies of the *Proceedings* for 1896 will send in their names to the Chairman, Dr. W. L. Williams, New York State Veterinary College, Ithaca, N. Y. An extra charge of fifty cents will be added for binding.

